# Depressive symptoms are associated with tumor necrosis factor alpha in systemic lupus erythematosus

**DOI:** 10.1186/s12974-015-0471-9

**Published:** 2016-01-06

**Authors:** Mariana Postal, Aline Tamires Lapa, Nailú Angélica Sinicato, Karina de Oliveira Peliçari, Fernando Augusto Peres, Lilian Tereza Lavras Costallat, Paula Teixeira Fernandes, Roberto Marini, Simone Appenzeller

**Affiliations:** Department of Medicine, Rheumatology Unit, Faculty of Medical Science State University of Campinas, Campinas, São Paulo CEP 13083-970 Brazil; Department of Pediatrics, Pediatric Rheumatology Unit, Faculty of Medical Science State University of Campinas, Campinas, São Paulo CEP 13083-970 Brazil; Department of Sport Sciences, Faculty of Physical Education State University of Campinas, Campinas, São Paulo CEP 13083-970 Brazil

**Keywords:** Tumor necrosis factor alpha (TNF-α), Depression, Anxiety, Systemic lupus erythematosus

## Abstract

**Background:**

Tumor necrosis factor alpha (TNF-α) is deeply related to pathogenesis of neurodevelopmental disorders, especially depression. The aim of this study was to explore potential relationships between sera TNF-α levels and mood and anxiety disorders in systemic lupus erythematosus (SLE) patients.

**Methods:**

We included 153 consecutive SLE patients (women 148; median age 30; range 10–62) and 40 (women 37; mean age 28.5; range 12–59) age- and sex-matched healthy controls. Mood and anxiety disorders were determined through Beck Depression and Beck Anxiety Inventory. SLE patients were further assessed for clinical and laboratory SLE manifestations. TNF-α levels were measured by enzyme-linked immunosorbent assay using commercial kits.

**Results:**

Depressive symptoms were identified in 70 (45.7 %) SLE patients and in 10 (25 %) healthy controls (*p* < 0.001). Anxiety symptoms were identified in 93 (60.7 %) SLE patients and in 16 controls (40 %) (*p* < 0.001). Sera TNF-α levels were increased in SLE patients with depressive symptoms (*p* < 0.001) and with anxiety symptoms (*p* = 0.014). A direct correlation between the severity of depressive symptoms and sera TNF-α levels (*r* = 0.22; *p* = 0.003) was observed. TNF-α levels were significantly increased in patients with active disease (*p* = 0.012). In addition, we observed a correlation between sera TNF-α levels and disease activity (*r* = 0.28; *p* = 0.008). In the multivariate analysis, sera TNF-α levels were independently associated with depressive symptoms (t = 3.28; 95 % CI 1.08–2.2; *p* = 0.002).

**Conclusions:**

Sera TNF-α levels are increased in SLE patients with mood and anxiety disorders. In SLE, sera TNF-α levels are independently associated with mood disorders. The etiology of mood disorders is still debated in SLE, but our findings suggest the presence of immunological basis for depression in SLE.

## Background

Systemic lupus erythematosus (SLE) is a chronic, inflammatory, immune-mediated disease with diverse clinical manifestations, affecting 0.1 % of the general population [1]. A large fraction of SLE patients demonstrate organic psychiatric and neurological disorders, indicating central nervous system (CNS) involvement [2]. Neuropsychiatric (NP) manifestations in SLE are diverse and may include major manifestations (i.e., stroke syndromes, severe organic brain syndrome, seizures, psychotic episodes, etc.) and minor abnormalities, including mood disorders and less severe cognitive deficits [2]. While the survival of SLE patients has increased substantially, such improvement, however, transforms into more damage accrual and disability that adversely affect the psychosocial health of SLE patients [3].

Depression and anxiety are commonly encountered in SLE patients [4, 5]. Some studies have found that greater disease activity, SLE severity, or longer disease duration increases vulnerability for clinical depression in SLE [4, 6–8]. Others have focused on physical disability and stress of living with a chronic disease as cause of depression in SLE [4, 5, 9, 10]. Although the majority of studies have focused on depressed mood or clinical unipolar depression in SLE, some authors suggest that symptoms of anxiety may be equally important in this cohort [8, 10, 11].

The pathogenesis of mood and anxiety disorder in SLE remains unclear. To date, studies aiming to investigate the relationship between mood disorders, anxiety, and proinflammatory cytokines have been scarce [12–15]. In the last two decades, since the initial reports of neural-immune interactions in depression, studies have shown a clear association between activation of the immune system, peripheral proinflammatory cytokines, and psychiatric symptoms [12]. The tumor necrosis factor alpha (TNF-α) is a pleiotropic cytokine that produces different stimuli in various physiological and pathological conditions [16]. TNF-α exerts its biological effect mainly by binding to tumor necrosis factor receptor 1 (TNFR1) and receptor 2 (TNFR2), causing activation of complex signaling cascades that mediate different intracellular effects. In the brain, TNFR1 seems to show a constitutive pattern of expression whereas TNFR2 is mainly expressed under stimulatory conditions [17]. TNF-α may underlie the mechanism of depression by an activation of the hypothalamo-pituitary-adrenocortical (HPA) axis, an activation of neuronal serotonin transporters and the stimulation of the indoleamine 2,3-dioxygenase which leads to tryptophan depletion [18].

Studies link peripheral cytokines and pathogenesis of depression in Alzheimer’s disease [17], in atypical depression [19], in major depressive disorder [19–21], and in multiple sclerosis [22, 23]. The aim of this study was to explore potential relationships between sera TNF-α levels and mood and anxiety disorders in SLE patients.

## Methods

### Design and subjects

Consecutive SLE patients followed at the Rheumatology Outpatient Clinic of State University of Campinas, between January 2010 and December 2011, were invited to participate in this cross-sectional study. Patients were included in the present study if they (i) fulfilled at least four criteria of American College of Rheumatology (ACR) [24] and (ii) had a follow-up duration of at least 6 months. Patients taking psychotropic medication were excluded from the study.

Healthy volunteers were included as a control group. The healthy controls were matched for age, sex, and demographic background. They were recruited from local community. Additionally, those control subjects who had histories of a psychiatric disorder other than depression diagnosed by a medical practitioner, or have been taking psychotropic medications, were also excluded.

### Ethics statement

This study was approved by the ethics committee at our institution (Faculty of Medical Sciences, State University of Campinas), and informed written consent was obtained from each participant and/or legal guardian.

### Clinical features

All patients had their medical histories and clinical and serological characteristics evaluated at study entry according to the ACR [24]. Features included in this protocol, such as age at onset of disease (defined as the age at which the first symptoms clearly attributable to SLE occurred), age at diagnosis (defined as the age when patients fulfilled four or more of the 1982 revised criteria for the classification of SLE) [24], and follow-up time were taken from medical charts. All clinical manifestations and laboratory findings were recorded at the day of blood withdrawal. Nephritis was diagnosed on the basis of proteinuria exceeding 0.5 g/L with abnormal urinary sediment and/or histological findings. Nephrotic syndrome was defined as proteinuria in excess of 3.0 g/day.

Hematological alterations were ascribed to lupus only in the absence of bone marrow suppression (leukopenia <4000 cells/mm^3^, thrombocytopenia <100,000 cells/mm^3^, hemolytic anemia). We also analyzed the presence of malar rash, discoid lesions, subacute cutaneous lesions, cutaneous vasculitis, photosensitivity, oral ulcers, arthritis, and serositis. Neurological and psychiatric involvement was defined according to ACR [25].

Treatment prescribed at the time of blood withdrawal, as well as its adverse events related to drug use, was recorded. Doses of oral and parenteral corticosteroids were analyzed and converted to the equivalent doses of prednisone.

### Laboratory features

Antinuclear antibodies (ANA) were determined by indirect immunoflurescence using HEp-2 cells as substrate and regarded as positive if higher than 1:40. Antidouble stranded DNA (dsDNA) antibodies were determined by indirect immunofluorescence using *Crithidia* as substrate and considered positive if higher than 1:10. Precipitating antibodies to extractable nuclear antigens (ENA) including Ro (SSA), La (SSB), and Sm were detected by a standardized enzyme-linked immunosorbent assay (ELISA) method and considered positive if higher than 1:40. Rheumatoid factor was detected by nephelometry and regarded as positive if higher than 10. Anticardiolipin antibodies (aCL) of the IgG and IgM isotypes were measured by an ELISA method [26]. The lupus anticoagulant (LA) activity was detected by coagulation assays in platelet-free plasma obtained by double centrifugation, following the recommendation of the subcommittee on LA of the Scientific and Standardization Committee of the International Society of Thrombosis and Homeostasis [27]. These measurements were carried out twice, at an interval of 12 weeks.

### Disease activity/cumulative damage evaluation

SLE patients were assessed for disease activity and cumulative damage. Disease activity was measured by the Systemic Lupus Erythematosus Disease Activity Index (SLEDAI) [28]. SLEDAI scores range between 0 and 105. Score of ≥3 was considered active disease [29]. Cumulative SLE-related damage in all patients was determined using the Systemic Lupus International Collaborating Clinics (SLICC)/ACR Damage Index (SDI) at the time of blood withdrawal. The range of SDI score varies from 0 to 47 [30].

### Blood samples

Nine milliliters of peripheral blood were drawn through venipuncture of the antecubital veins in all subjects. Serum was obtained by centrifugation (3000 rpm for 15 min), and separated sera were kept in aliquots at −80 °C until the time of assay. None of the samples were taken during an episode of acute or chronic infection because TNF-α could be increased due to a secondary cause [31]. Commercially available kits from R&D Systems (London, UK) were used for the measurement of sera TNF-α levels by ELISA, carried out in accordance with the manufacturer’s instructions.

### Mood and anxiety evaluation

To assess clinically symptoms of depression and anxiety, the Beck Depression Inventory (BDI) [32, 33] and Beck Anxiety Inventory (BAI) [34, 35] were used. Validated Brazilian Portuguese version of BDI and BAI was applied [33, 35]. All the participants answered both inventories. These scales consist of 21 items, each describing a common symptom of depression/anxiety. The respondent is asked to rate how much he or she has been bothered by each symptom over the past month on a 4-point scale ranging from 0 to 3. The items are summed to obtain a total score that can range from 0 to 63. The cutoffs used for the BDI are 0–13, no/minimal depression; 14–19, mild depression; 20–28, moderate depression; and 29–63, severe depression and for the BAI are 0–7, no/minimal level of anxiety; 8–15, mild anxiety; 16–25, moderate anxiety; and 26–63, severe anxiety.

### Statistical analysis

We performed normality tests. Our data did display a non-parametric distribution in the Shapiro-Wilk test; thus, we used the Mann-Whitney *U* test for comparison of TNF-α and independent groups (i.e., groups (SLE patients/controls), disease activity (SLEDAI ≥ 3/SLEDAI < 3), cumulative damage (SDI ≥ 1/SDI = 0), sex (male/female), and medication (taking any medication/without medication). The correlations between depressive/anxiety symptoms and TNF-α were explored by Spearman’s rank correlation. Multivariate analysis was performed including age, SLE duration, disease activity, and cumulative damage, severity of depression, and anxiety. TNF-α was used as a dependent variable. The level of significance was set to *p* < 0.05. Statistical analysis was performed using SPSS® 21.0.

## Results

### Demographics and clinical features

We included 153 consecutive SLE patients. One hundred forty-eight (96.73 %) were female with median age of 30 years (range 10–62). The median of disease duration was 9 years (range 0–33 years). The control group consisted of 40 healthy controls (37 women) with a median age of 28.5 years (range 12–59 years). Patients and healthy controls were statistically comparable in terms of age and sex (Table 1).

**Table 1:** Demographic and clinical features of SLE patients and healthy controls at study entry

	SLE patients *N* = 153 (%)	Healthy controls *N* = 40 (%)
Sex		
Female	148 (96.7)	37 (92.5)
Age (years)	30 (range 10–62)	28.5 (range 12–59)
Disease duration (years)	9 (range 0–33)	–
SLEDAI score		
SLEDAI ≥3 *N* = 68	8 (range 4–18)	–
SLEDAI <3 *N* = 85	0 (range 0–2)	
SDI ≥1 *N* = 77	1 (range 0–9)	–

Results were given as median and range

At the time of study entry, 68 (44.4 %) SLE patients had active disease (SLEDAI ≥3) with median SLEDAI scores of 8 (range 2–18). The 85 (55.6 %) inactive patients had a median SLEDAI score of 0 (range 0–2). At the same time, 77 (50.32 %) SLE patients had cumulative damage (SDI >1) and median SDI scores of 1 (range 0–9).

Depressive symptoms were identified in 70 (45.7 %) SLE patients and in 10 (25 %) healthy controls (*p* < 0.001). The mean of BDI scores in SLE patients was 15.08 ± 13.02 (range 0–62) and 4.68 ± 4.27 (range 0–16) in controls. The mean of BAI scores was 15.92 ± 15.25 (range 0–63) in SLE patients and 6.88 ± 5.84 (range 0–21) in controls. Anxiety symptoms were observed in 93 (60.7 %) in SLE patients and in 16 (40 %) controls (*p* < 0.001). The extent of depressive and anxiety symptoms is described in Table 2.We did not observe an association between disease activity and depression (*p* = 0.22) or anxiety (*p* = 0.88).

**Table 2:** Percentage of all individuals included in the study, according the severity of depression and anxiety symptoms

Severity	Depression SLE patients (%)	Depression controls (%)	Anxiety SLE patients (%)	Anxiety controls (%)
Mild	23 (32.8)	10 (24.4)	29 (31.2)	11 (64.7)
Moderate	19 (27.1)	0	37 (39.8)	5 (29.4)
Severe	28 (40)	0	29 (31.2)	0
Total	70 (45.7)	10 (25)	93 (60.7)	16 (40)

### Cytokine assay

The median of TNF-α levels was 1.93 pg/mL (range 0.45–11.17) in SLE patients compared to 1.50 pg/mL (range 0.39–6.35) in healthy controls (*p* = 0.003). The median of TNF-α levels in SLE patients with depressive symptoms was 2.42 (range 0.45–11.17) versus 1.45 (range 0.57–3.23) in SLE patients without depressive symptoms (*p* < 0.001). The median in SLE patients with anxiety symptoms was 2.15 (range 0.45–11.17) versus 1.59 (0.7–3.23) in SLE patients without anxiety symptoms (*p* = 0.014). A direct correlation between sera TNF-α levels and the severity of depression symptoms (*r* = 0.51; *p* < 0.001) was observed. All Spearman rank correlation analyses between TNF-α and other variables are described in Table 3.

**Table 3:** Spearman rank correlation between TNF-α and clinical/treatment variables

	Variables	Spearman’s correlation coefficient	*p* value
	Clinical		
TNF-α	BDI	0.51	<0.001*
	BAI	0.06	0.45
	SLEDAI	0.28	0.008*
	SDI	−0.18	0.86
	Treatment		
	Corticosteroids	0.15	0.15
	Chloroquine	−0.06	0.52
	Immunosuppressants	−0.005	0.96

*p < 0.05

TNF-α levels were further significantly increased in patients with active disease (*p* = 0.012). In addition, we observed a correlation between sera TNF-α levels and disease activity (*r* = 0.28; *p* = 0.008). No association between TNF-α levels and other clinical, laboratory variable, SDI scores, and current medication was observed. No difference in TNF-α levels was observed between patients with and without hydroxychloroquine or other immunosuppressants. In the multivariate analysis, sera TNF-α levels were independently associated with depressive symptoms (*t* = 3.28; 95 % CI 1.08–2.2; *p* = 0.002).

## Discussion

Substantial evidences implicate inflammation as a critical mediator in the pathophysiology of mood and anxiety disorders. Peripheral cytokines produced during the inflammatory response may be useful biomarkers when investigating the potential relationship between inflammation and mood and anxiety disorders [36]. We aimed to explore potential relationships between sera TNF-α levels and mood and anxiety disorders in SLE patients.

There are animal models and human studies that link peripheral cytokine and inflammatory responses signal in the brain [37–43]. Several studies have investigated the role of inflammation in depression [43–45]. Animal studies have shown that injection of proinflammatory cytokines in rodents result in a sickness behavior, including decreased social exploration, decreased appetite, and decreased activities [44].

Depression and sickness behavior may be the result of many physiological stressors in life [44]. We observed a direct correlation between sera TNF-α levels and the severity of depression symptoms. Sickness behavior has been proved to be mediated by proinflammatory cytokines, such as interleukin (IL)-1 and IL-6 and TNF-α [46]. These inflammatory cytokines interact with practically every pathophysiologic domain relevant to depression, including neurotransmitter metabolism, neuroendocrine function, and synaptic plasticity [19].

There are, at least, more three mechanisms that link the activation of the cytokine system, of which TNF-α is a part, to the pathophysiology of depression [18]. First, the activation of the cytokine system might play a causative role in the depression-related activation of the HPA axis [14, 47, 48]. The stress response system is intricately linked with proinflammatory signaling. The release of TNF-α and interleukin 6 have been shown to increase the levels of corticotropin-releasing hormone (CRH), adrenocorticotropic hormone (ACTH), and cortisol which act directly on hypothalamic and pituitary cells [49–53]. The upregulation of the HPA axis is an important finding associated with depression [54, 55], highlighting the potential for direct clinical significance of raised proinflammatory cytokines, mainly TNF-α [12].

Second, TNF-α activates neuronal serotonin transporters [43, 47]. During the process of depression, the uptake of serotonin is diminished. Drugs like selective serotonin reuptake inhibitors (SSRI) are used in the therapy of depression because SSRIs lead to recovery from depression via deactivation of serotonin transporters [47]. One study demonstrated that TNF-α stimulated serotonin uptake in both a rat embryonic raphe cell line and in mouse midbrain and striatal synaptosomes. These results provided evidence that TNF-α can acutely regulate neuronal serotonin transporter activity [43].

Third, TNF-α stimulates indoleamine 2,3-dioxygenase (IDO) leading to tryptophan depletion. The activation of IDO by TNF-α additionally leads to the production of glutamatergic agonists. The role of increased glutamatergic neurotransmission in the pathogenesis of depression remains inconclusive [56, 57]. The higher consumption of serotonin and its precursor tryptophan due to IDO activation may justify the reduced availability of serotonin and link TNF-α to depression [14, 47].

In addition, the relation between TNF-α and depression in SLE patients may be explained by the long-term use of corticosteroids. The hypothesis of depression as a stress-related disorder is that chronic stress derived from long-term use of corticosteroids impairs corticosteroid receptor signaling [58]. Corticotrophin-releasing hormone and arginine vasopressin are the main drivers of the stress hormone system that elicit corticotrophin into the periphery and thereby activate the corticosteroid release from the adrenal cortex [58]. A severe clinical condition and an inadequate adaptation to stress can appear when these stress hormones are persistently hypersecreted [58]. There was no association between corticosteroids and TNF-α in our cohort. In fact, we did not evaluate long-term use of corticosteroids; we just evaluated current medication dosage. Patients with autoimmune disease have higher levels of proinflammatory cytokines [59–61]; consequently, they have a more expressive interaction. Although these inflammatory theory of depression have been studied in depression in Alzheimer’s disease [17], in atypical depression [19], in major depressive disorder [19–21], and in multiple sclerosis [22, 23], these mechanisms have not been elucidated in SLE so far.

In this study, we observed higher sera levels of TNF-α in individuals with depression and with anxiety. We observed that sera TNF-α levels in SLE patients were positively correlated with the severity of depression. Another cross-sectional study supports our finding. The data included 54 SLE patients and 54 healthy controls, and higher sera TNF-α was associated with more severe depressive symptoms [59]. These findings suggest a potential role of TNF-α on depressive symptoms in SLE patients.

Our data shows higher prevalence of depression and anxiety in SLE patients compared to healthy controls. High prevalence of mood and anxiety disorders in SLE patients is in accordance with estimates reported in previous studies [3, 6, 62, 63]. Comparative studies of depression and anxiety between SLE and other disorders are limited by their relatively small sample sizes, comparison with relatively rare inflammatory diseases and the lack of matched healthy controls with background prevalence of depression and anxiety for comparison [64–66].

Given the elevated rates of depressive and anxiety disorders observed in SLE patients, it is important to consider some crucial factors. Disease activity and severity in SLE may contribute to psychiatric symptomatology through shared pathophysiological mechanisms, including antineuronal and antiphospholipid antibodies [67], and proinflammatory cytokines [68]. In addition, disease duration and CNS complications may increase vulnerability for psychiatric disorders in SLE patients [6].

We observed that TNF-α levels were significantly increased in patients with active disease. Previous studies reported an association between this cytokine and disease activity [60, 69–72], suggesting that TNF-α could be a potential biomarker to predict flares in SLE patients. However, in our cohort, we did not observe an association between disease activity and depression/anxiety. Our data corroborates previous studies that did not observe an association between depressive and anxiety disorders and disease activity in SLE patients [4, 7]. Previous studies have associated symptoms of depression and anxiety with physical disability and stress of living with a chronic disease [5]. Psychological distress may be associated with SLE outcomes, including fatigue [73], physical disability [74], and decreased cognitive functioning [75]. Mood disorder and anxiety might be secondary to SLE activity or to psychological distress [4]. Longitudinal studies are necessary to determine the possible role of each component in relation to cause-effect.

It is important to consider some limitations of the study analyzing depression mediated by TNF-α, such as the small number of healthy controls included and the short follow-up period. Longitudinal studies are necessary to determine the causal relationship between TNF-α and depression in SLE patients.

## Conclusions

In summary, sera TNF-α levels are increased in SLE patients with mood and anxiety disorders. In SLE, sera TNF-α levels were independently associated with depression and with disease activity. The etiology of mood disorders is still debated in SLE, but our findings suggest the presence of immunological basis for depression in SLE patients.

## Abbreviations

aCL: anticardiolipin
ACR: American College of Rheumatology
ACTH: adrenocorticotropic hormone
ANA: antinuclear antibodies
BAI: Beck Anxiety Inventory
BDI: Beck Depression Inventory
CNS: central nervous system
CRH: corticotropin-releasing hormone
dsDNA: antidouble stranded DNA
ELISA: enzyme-linked immunosorbent assay
ENA: extractable nuclear antigens
HPA: hypothalamo-pituitary-adrenocortical
IDO: indoleamine 2,3-dioxygenase
IL: interleukin
LA: lupus anticoagulant
NP: neuropsychiatric
SDI: Systemic Lupus International Collaborating Clinics (SLICC)/ACR Damage Index
SLE: systemic lupus erythematosus
SLEDAI: Systemic Lupus Erythematosus Disease Activity Index
SSRI: selective serotonin reuptake inhibitors
TNFR1: tumor necrosis factor receptor 1
TNFR2: tumor necrosis factor receptor 2
TNF-α: tumor necrosis factor alpha

## References

[1] Postal M, Costallat LT, Appenzeller S (2011). Neuropsychiatric manifestations in systemic lupus erythematosus: epidemiology, pathophysiology and management. CNS Drugs.

[2] Hanly JG, Harrison MJ (2005). Management of neuropsychiatric lupus. Best Pract Res Clin Rheumatol.

[3] Mak A, Tang CS, Chan MF, Cheak AA, Ho RC (2011). Damage accrual, cumulative glucocorticoid dose and depression predict anxiety in patients with systemic lupus erythematosus. Clin Rheumatol.

[4] Jarpa E, Babul M, Calderón J, González M, Martínez ME, Bravo-Zehnder M (2011). Common mental disorders and psychological distress in systemic lupus erythematosus are not associated with disease activity. Lupus..

[5] Seawell AH, Danoff-Burg S (2004). Psychosocial research on systemic lupus erythematosus: a literature review. Lupus.

[6] Bachen EA, Chesney MA, Criswell LA (2009). Prevalence of mood and anxiety disorders in women with systemic lupus erythematosus. Arthritis Rheum.

[7] Nery FG, Borba EF, Viana VS, Hatch JP, Soares JC, Bonfá E (2008). Prevalence of depressive and anxiety disorders in systemic lupus erythematosus and their association with anti-ribosomal P antibodies. Prog Neuropsychopharm Biol Psych..

[8] Seguí J, Ramos-Casals M, García-Carrasco M, de Flores T, Cervera R, Valdés M (2000). Psychiatric and psychosocial disorders in patients with systemic lupus erythematosus: a longitudinal study of active and inactive stages of the disease. Lupus..

[9] Shortall E, Isenberg D, Newman SP (1995). Factors associated with mood and mood disorders in SLE. Lupus.

[10] Waterloo K, Omdal R, Husby G, Mellgren SI (1998). Emotional status in systemic lupus erythematosus. Scandanavian J Rheumatol..

[11] Slattery MJ, Dubbert BK, Allen AJ, Leonard HL, Swedo SE, Gourley MF (2004). Prevalence of obsessive-compulsive disorder in patients with systemic lupus erythematosus. J Clin Psychiatry..

[12] Dowlati Y, Herrmann N, Swardfager W, Liu H, Sham L, Reim EK (2010). A meta-analysis of cytokines in major depression. Biol Psychiatry..

[13] Liu Y, Ho RC, Mak A (2012). The role of interleukin (IL)-17 in anxiety and depression of patients with rheumatoid arthritis. Int J Rheum Dis.

[14] Himmerich H, Fulda S, Linseisen J, Seiler H, Wolfram G, Himmerich S (2008). Depression, comorbidities and the TNF-alpha system. Eur Psychiatry..

[15] Zunszain PA, Hepgul N, Pariante CM (2013). Inflammation and depression. Curr Top Behav Neurosci.

[16] Postal M, Appenzeller S (2011). The role of tumor necrosis factor-alpha (TNF-α) in the pathogenesis of systemic lupus erythematosus. Cytokine.

[17] Baud V, Karin M (2001). Signal transduction by tumor necrosis factor and its relatives. Trends Cell Biol.

[18] Krishnadas R, Cavanagh J (2012). Depression: an inflammatory illness?. J Neurol Neurosurg Psychiatry.

[19] Khairova RA, Machado-Vieira R, Du J, Manji HK (2009). A potential role for pro-inflammatory cytokines in regulating synaptic plasticity in major depressive disorder. Int J Neuropsychopharmacol.

[20] Kaster MP, Gadotti VM, Calixto JB, Santos AR, Rodrigues AL (2012). Depressive-like behavior induced by tumor necrosis factor-α in mice. Neuropharmacology.

[21] Clark IA, Alleva LM, Vissel B (2010). The roles of TNF in brain dysfunction and disease. Pharmacol Ther.

[22] Kollias G, Douni E, Kassiotis G, Kontoyiannis D (1999). The function of tumor necrosis factor and receptors in models of multi-organ inflammation, rheumatoid arthritis, multiple sclerosis and inflammatory bowel disease. Ann Rheum Dis.

[23] Mikova O, Yakimova R, Bosmans E, Kenis G, Maes M (2001). Increased serum tumor necrosis factor alpha concentrations in major depression and multiple sclerosis. Eur Neuropsychopharmacol.

[24] Tan EM, Cohen AS, Fries JF, Masi AT, McShane DJ, Rothfield NF (1982). The 1982 revised criteria for the classification of systemic lupus erythematosus. Arthritis Rheum..

[25] ACR Ad Hoc Committee on Neuropsychiatric Lupus Nomenclature (1999). The American College of Rheumatology nomenclature and case definitions for neuropsychiatric lupus syndromes. Arthritis Rheum.

[26] Harris EN, Gharavi AE, Patel SP, Hughes GR (1987). Evaluation of the anti-cardiolipin antibody test: report of an international workshop held 4 April 1986. Clin Exp Immunol.

[27] Brandt JT, Triplett DA, Alving B, Scharrer I (1995). On behalf of the Subcommittee on Lupus Anticoagulant/Antiphospholipid Antibody of the Scientific and Standardization Committee of the ISTH. Criteria for the diagnosis of lupus anticoagulants: an update. Thromb Haemost.

[28] Bombardier C, Gladman DD, Urowitz MB, Caron D, Chang CH (1992). Derivation of the SLEDAI. A disease activity index for lupus patients. The Committee on Prognosis Studies in SLE. Arthritis Rheum.

[29] Yee CS, Farewell VT, Isenberg DA, Griffiths B, Teh LS, Bruce IN (2011). The use of Systemic Lupus Erythematosus Disease Activity Index-2000 to define active disease and minimal clinically meaningful change based on data from a large cohort of systemic lupus erythematosus patients. Rheumatology (Oxford)..

[30] Gladman DD, Urowitz MB, Goldsmith CH, Fortin P, Ginzler E, Gordon C (1997). The reliability of the Systemic Lupus International Collaborating Clinics/American College of Rheumatology Damage Index in patients with systemic lupus erythematosus. Arthritis Rheum..

[31] Galley HF, Webster NR (1996). The immuno-inflammatory cascade. Br J Anaesth.

[32] Beck AT, Ward CH, Mendelson M, Mock J, Erbaugh J (1961). An inventory for measuring depression. Arch Gen Psychiatry.

[33] Gomes-Oliveira MH, Gorenstein C, Lotufo Neto F, Andrade LH, Wang YP (2012). Validation of the Brazilian Portuguese version of the Beck Depression Inventory-II in a community sample. Rev Bras Psiquiatr.

[34] Beck AT, Epstein N, Brown G, Steer RA (1988). An inventory for measuring clinical anxiety: psychometric properties. J Consult Clin Psychol.

[35] Quintão S, Delgado AR, Prieto G (2013). Validity study of the Beck Anxiety Inventory (Portuguese version) by the Rasch Rating Scale model. Psicol Reflex Crit.

[36] Rosenblat JD, Cha DS, Mansur RB, McIntyre RS (2014). Inflamed moods: a review of the interactions between inflammation and mood disorders. Prog Neuropsychopharmacol Biol Psychiatry.

[37] Tuglu C, Kara SH, Caliyurt O, Vardar E, Abay E (2003). Increased serum tumor necrosis factor-alpha levels and treatment response in major depressive disorder. Psychopharmacology (Berl).

[38] Marchi N, Rasmussen P, Kapural M, Fazio V, Kight K, Mayberg MR (2003). Peripheral markers of brain damage and blood-brain barrier dysfunction. Restor Neurol Neurosci..

[39] Kaster MP, Gadotti VM, Calixto JB, Santos AR, Rodrigues AL (2012). Depressive-like behavior induced by tumor necrosis factor-a in mice. Neuropharmacology.

[40] Krügel U, Fischer J, Radicke S, Sack U, Himmerich H (2013). Antidepressant effects of TNF-α blockade in an animal model of depression. J Psychiatr Res.

[41] Chen J, Song Y, Yang J, Zhang Y, Zhao P, Zhu XJ (2013). The contribution of TNF-α in the amygdala to anxiety in mice with persistent inflammatory pain. Neurosci Lett..

[42] Karson A, Demirtaş T, Bayramgürler D, Balci F, Utkan T (2013). Chronic administration of infliximab (TNF-α inhibitor) decreases depression and anxiety-like behaviour in rat model of chronic mild stress. Basic Clin Pharmacol Toxicol.

[43] Zhu CB, Blakely RD, Hewlett WA (2006). The proinflammatory cytokines interleukin-1beta and tumor necrosis factor-alpha activate serotonin transporters. Neuropsychopharmacology.

[44] Lu S, Peng H, Wang L, Vasish S, Zhang Y, Gao W (2013). Elevated specific peripheral cytokines found in major depressive disorder patients with childhood trauma exposure: a cytokine antibody array analysis. Compr Psychiatry..

[45] Camacho A (2013). Is anxious-depression an inflammatory state?. Med Hypotheses.

[46] Dantzer R (2009). Cytokine, sickness behavior, and depression. Immunol Allergy Clin North Am.

[47] Berthold-Losleben M, Himmerich H (2008). The TNF-alpha system: functional aspects in depression, narcolepsy and psychopharmacology. Curr Neuropharmacol.

[48] Doczy EJ, Seroogy K, Harrison CR, Herman JP (2009). Hypothalamo-pituitary-adrenocortical axis, glucocorticoids, and neurologic disease. Immunol Allergy Clin North Am.

[49] Black PH (1994). Immune system-central nervous system interactions: effect and immunomodulatory consequences of immune system mediators on the brain. Antimicrob Agents Chemother.

[50] Dantzer R, Wollman E, Vitkovic L, Yirmiya R (1999). Cytokines and depression: fortuitous or causative association?. Mol Psychiatry.

[51] Vallieres L, Rivest S (1999). Interleukin-6 is a needed proinflammatory cytokine in the prolonged neural activity and transcriptional activation of corticotropin-releasing factor during endotoxemia. Endocrinology.

[52] Chrousos GP (1995). The hypothalamic-pituitary-adrenal axis and immune-mediated inflammation. N Engl J Med.

[53] McCann SM, Lyson K, Karanth S, Gimeno M, Belova N, Kamat A (1995). Mechanism of action of cytokines to induce the pattern of pituitary hormone secretion in infection. Ann N Y Acad Sci..

[54] Cowen PJ (2002). Cortisol, serotonin and depression: all stressed out?. Br J Psychiatry.

[55] Young EA, Haskett RF, Grunhaus L, Pande A, Weinberg VM, Watson SJ (1994). Increased evening activation of the hypothalamic-pituitary-adrenal axis in depressed patients. Arch Gen Psychiatry..

[56] Müller N, Schwarz MJ (2007). Immunological aspects of depressive disorders. Nervenarzt.

[57] Wichers M, Maes M (2002). The psychoneuroimmuno-pathophysiology of cytokine-induced depression in humans. Int J Neuropsychopharmacol.

[58] Lang UE, Borgwardt S (2013). Molecular mechanisms of depression: perspectives on new treatment strategies. Cell Physiol Biochem.

[59] Mak A, Tang C, Ho R (2013). Serum tumour necrosis factor-alpha is associated with poor health-related quality of life and depressive symptoms in patients with systemic lupus erythematosus. Lupus.

[60] Postal M, Peliçari KO, Sinicato NA, Marini R, Costallat LT, Appenzeller S (2013). Th1/Th2 cytokine profile in childhood-onset systemic lupus erythematosus. Cytokine..

[61] Eijsbouts AM, van den Hoogen FH, Laan RF, Hermus AR, Sweep CG, van de Putte LB (2005). Hypothalamic-pituitary-adrenal axis activity in patients with rheumatoid arthritis. Clin Exp Rheumatol..

[62] Campbell R, Cooper GS, Gilkeson GS (2008). Two aspects of the clinical and humanistic burden of systemic lupus erythematosus: mortality risk and quality of life early in the course of disease. Arthritis Rheum.

[63] Ruperto N, Buratti S, Duarte-Salazar C, Pistorio A, Reiff A, Bernstein B (2004). Health-related quality of life in juvenile-onset systemic lupus erythematosus and its relationship to disease activity and damage. Arthritis Rheum..

[64] Lim L, Ron MA, Ormerod IE, David J, Miller DH, Logsdail SJ (1988). Psychiatric and neurological manifestations in systemic lupus erythematosus. Q J Med..

[65] Harboe E, Tjensvoll AB, Maroni S, Gøransson LG, Greve OJ, Beyer MK (2009). Neuropsychiatric syndromes in patients with systemic lupus erythematosus and primary Sjögren syndrome: a comparative population-based study. Ann Rheum Dis..

[66] Líndal E, Thorlacius S, Steinsson K, Stefánsson JG (1995). Psychiatric disorders among subjects with systemic lupus erythematosus in an unselected population. Scand J Rheumatol.

[67] Zandman-Goddard G, Chapman J, Shoenfeld Y (2007). Autoantibodies involved in neuropsychiatric SLE and antiphospholipid syndrome. Semin Arthritis Rheum.

[68] Zorrilla EP, Luborsky L, McKay JR, Rosenthal R, Houldin A, Tax A (2001). The relationship of depression and stressors to immunological assays: a meta-analytic review. Brain Behav Immun..

[69] Sabry A, Sheashaa H, El-Husseini A, Mahmoud K, Eldahshan KF, George SK (2006). Proinflammatory cytokines (TNF-alpha and IL-6) in Egyptian patients with SLE: its correlation with disease activity. Cytokine..

[70] Gabay C, Cakir N, Moral F, Roux-Lombard P, Meyer O, Dayer JM (1997). Circulating levels of tumor necrosis factor soluble receptors in systemic lupus erythematosus are significantly higher than in other rheumatic diseases and correlate with disease activity. J Rheumatol..

[71] Mahmoud RA, El-Gendi HI, Ahmed HH (2005). Serum neopterin, tumor necrosis factor-alpha and soluble tumor necrosis factor receptor II (p75) levels and disease activity in Egyptian female patients with systemic lupus erythematosus. Clin Biochem.

[72] Studnicka-Bencke A, Steiner G, Petera P (1996). Tumor necrosis factor alpha and its soluble receptors parallel clinical disease and autoimmune activity in systemic lupus erythematosus. Br J Rheumatol.

[73] McKinley PS, Ouellette SC, Winkel GH (1995). The contributions of disease activity, sleep patterns, and depression to fatigue in systemic lupus erythematosus: a proposed model. Arthritis Rheum.

[74] Ward MM, Lotstein DS, Bush TM, Lambert RE, van Vollenhoven R, Neuwelt CM (1999). Psychosocial correlates of morbidity in women with systemic lupus erythematosus. J Rheumatol..

[75] Da Costa D, Dobkin PL, Pinard L, Fortin PR, Danoff DS, Esdaile JM (1999). The role of stress in functional disability among women with systemic lupus erythematosus: a prospective study. Arthritis Care Res..

